# TRPA1, TRPV1, and Caffeine: Pain and Analgesia

**DOI:** 10.3390/ijms25147903

**Published:** 2024-07-19

**Authors:** Elizabeth A. Puthumana, Luna Muhamad, Lexi A. Young, Xiang-Ping Chu

**Affiliations:** Departments of Biomedical Sciences, School of Medicine, University of Missouri-Kansas City, Kansas City, MO 64108, USA; eapk9d@umsystem.edu (E.A.P.); lamvdz@umkc.edu (L.M.); lyfzt@umkc.edu (L.A.Y.)

**Keywords:** caffeine, methylxanthine, TRP, TRPV1, TRPA1, pain, analgesia

## Abstract

Caffeine (1,3,7-trimethylxanthine) is a naturally occurring methylxanthine that acts as a potent central nervous system stimulant found in more than 60 different plants and fruits. Although caffeinated beverages are widely and casually consumed, the application of caffeine beyond dietary levels as pharmacologic therapy has been recognized since the beginning of its recorded use. The analgesic and vasoactive properties of caffeine are well known, but the extent of their molecular basis remains an area of active research. There is existing evidence in the literature as to caffeine’s effect on TRP channels, the role of caffeine in pain management and analgesia, as well as the role of TRP in pain and analgesia; however, there has yet to be a review focused on the interaction between caffeine and TRP channels. Although the influence of caffeine on TRP has been demonstrated in the lab and in animal models, there is a scarcity of data collected on a large scale as to the clinical utility of caffeine as a regulator of TRP. This review aims to prompt further molecular research to elucidate the specific ligand–host interaction between caffeine and TRP by validating caffeine as a regulator of transient receptor potential (TRP) channels—focusing on the transient receptor potential vanilloid 1 (TRPV1) receptor and transient receptor potential ankyrin 1 (TRPA1) receptor subtypes—and its application in areas of pain.

## 1. Introduction

Transient receptor potential (TRP) channels were initially identified in 1989 as a novel protein channel in the Drosophila species [1]. In subsequent years, TRP channels have been distinguished as a unique and diverse superfamily of cation channels. TRP channels are known to assemble as homo- or heterotetramers with a six-transmembrane helix topology (S1–S6) and two cytoplasmic domains (NH_2_ and COOH termini) [2,3,4,5]. The transmembrane core of each TRP subunit contains a voltage sensor domain and a pore-forming domain between the last two transmembranes (S5–S6) [2,3]. The cytoplasmic end of the S6 helix forms the lower gate that opens and closes to regulate cation entry into the channel [3]. Subfamilies of TRP channels have varying amounts of amino, carboxyl termini, and amino acid sequences that are located intracellularly [6]. The subfamilies of TRP channel proteins are divided depending on respective amino acid sequences [7].

TRP activation is polymodal in nature, as TRP channels are stimulated and regulated by numerous stimuli that are expressed throughout the body, including signals PIP2, Ca^2+^, exogenous ligands, second messengers, reactive oxygen–nitrogen species, and physical stimuli [5,8,9,10,11]. Voltage-gated activation is the best-understood form of activation in TRP channels [8]. For example, TRPV1 is known to have a gating charge of 0.5 to 0.7 e0 [12]. Activation via voltage-gated ion channels involves a series of positively charged amino acids in the fourth transmembrane segment (S4), voltage-gated K^+^, Na^+^, and Ca^2+^ channels. The S4 senses changes in the electric field in the plasma membrane due to the force in the field; these changes in the transmembrane voltage drive the movement of S4, which is coupled with the opening of the activation gate [10,11,12,13]. The activation of TRP channels changes the membrane potential, translocates signaling across the cell membrane, and alters enzymatic activity, which triggers downstream pathways [7,14]. Multiple signaling pathways are affected by TRP activation including the mitogen-activated protein kinase (MAPK) pathway, nuclear factor kappa-B (NF-kB) pathway, and AMP-activated protein kinase (AMPK) pathway [7,8,9,10,11,12,13,14,15,16,17,18,19,20]. 

TRP superfamilies have been described in the literature as playing a role in many areas of human pathophysiology, including pain, thermoregulation, pruritus, chemoreception, metabolic regulation, immunomodulation including cytokine production, stroke, vasospasm, migraine, temperature regulation, and epilepsy [21,22,23,24,25,26,27,28,29,30,31,32,33,34,35]. 

Caffeine’s influence on TRP channels, particularly in pain management and analgesia, has been suggested in the existing literature. Studies have established the effects of caffeine on TRP channels, and the role of TRP channels in pain modulation is well documented. However, no comprehensive review has specifically addressed the interaction between caffeine and TRP channels. While laboratory and animal model studies indicate that caffeine affects TRP channels, there are limited large-scale clinical data on the therapeutic potential of caffeine as a TRP channel regulator. A scoping review is appropriate for this topic, as it aims to map the existing literature on a broad topic to identify key concepts, gaps in research, and types of evidence available. The review questions and objectives are exploratory and intended to clarify the potential of caffeine as a regulator of TRP channels, particularly focusing on the TRPV1 and TRPA1 receptor subtypes and their implications in pain management. 

## 2. Methodology

Keywords “caffeine”, “methylxanthine”, “TRP”, “TRPA1”, “TRPV1”, “pain”, and “analgesia” were entered into PubMed and Google Scholar databases for an initial literature search. Greater than fifty percent of articles cited were indicated as published within the most recent ten years to ensure inclusion of recent and relevant information. Articles prior to this were included if containing foundational information relevant to the topic. Journal articles yielding results from “caffeine” and “TRP” searches regarding the interaction between caffeine and tryptophan (Trp) were excluded. A total of 141 resources were found to have relevance to either caffeine structure, function, and mechanism of action; caffeine interaction with adenosine receptors; caffeine in analgesia; TRP channels in various roles; TRPA1 structure, function, and role in analgesia; TRPA1 interaction with caffeine; TRPV1 structure, function, and role in analgesia. The most recent literature search was conducted on 15 June 2024. As a scoping review that summarizes what is known about caffeine–TRP interaction, this review may act to prompt further research into the role of caffeine in the rapidly growing area of TRP-targeting drug therapies.

## 3. TRP Channel Overview

### 3.1. TRPV1 and TRPA1: Central Nervous System (CNS) Distribution

Various TRP channels are found to have high levels of expression in the central nervous system (CNS) with involvement in a wide range of pathologies, including migraine headache, epilepsy, pain, and inflammation [26,27,36,37,38,39,40,41,42]. Moreover, suppression of TRPC channel degradation prevents neuronal cell death in experimental strokes, further highlighting the crucial importance of TRP channels in CNS function [26,27].

**Figure 1:**
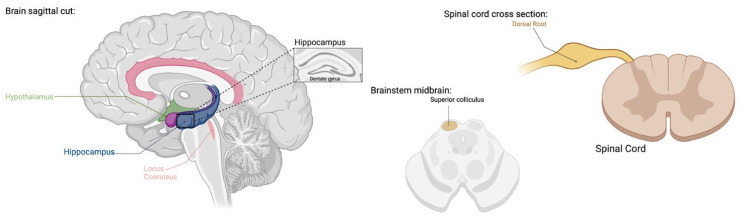
TRPV1 expression has been found in dorsal root ganglia, hippocampus, cortex, hypothalamus, olfactory nuclei, dentate gyrus, locus coeruleus, superior colliculus, and spinal cord [22,37,38,39,43,44,45,46]. TRPV1 functions include pain, inflammation, behavior, glial function, neuronal function, synaptic transmission, plasticity, and neurodegeneration [26,27,36,37,38,39,40,41,42]. (Created with BioRender.com).

TRPV1 expression has been confirmed in both C-fibers and Aσ-fibers that project to the dorsal horn with channels present in 70% of small cervical DRG neurons as well as distribution in trigeminal and vagal afferents [22,43,44] (Figure 1). TRP channels have been shown to play a role in migraine headaches through expression in the trigeminal system and other brain regions involved in the pathophysiology of migraines, as well as their interaction with neuropeptides involved in the development of migraine attacks [36]. Although a large body of early research describes TRPV1 to be most abundantly present in trigeminal nodose ganglia, more recent experiments using immunohistochemistry, in situ hybridization, RT-PCR, and capsaicin response have revealed the presence of TRPV1 on a wider scale in the CNS in both human and animal models [45,46]. Outside of the dorsal root ganglion, TRPV1 expression is prominent in the cortex and hippocampus but has also been found in a variety of subcortical areas [37,38] (Figure 1). TRPV1 has been found to play variable roles in pain and inflammation, behavior, glial function, neuronal function, synaptic transmission, plasticity, and neurodegeneration through CNS involvement [37,38,39].

**Figure 2:**
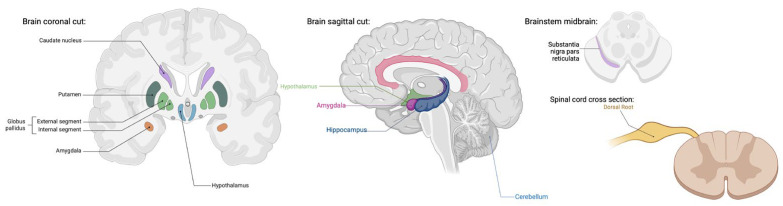
TRPA1 expression has been found in the cortex, caudate nucleus, putamen, globus pallidus, substantia nigra, hippocampus, cerebellum, amygdala, hypothalamus, dorsal root, vagal and trigeminal ganglion, and glial cells [9,47,48,49,50,51]. TRPA1 functions include pain, inflammation, and neuronal regulation [9,47,48,49,50]. (Created with BioRender.com).

TRPA1 channels are widely distributed in the human body in both excitable and non-excitable cell types. A substantial portion of primary sensory neurons in the dorsal root, vagal, and trigeminal ganglia, as well as glial cells (astrocytes, oligodendrocytes, and Schwann cells) have been found to be TRPA1-positive with functions in pain, inflammation, and neural regulation [9,47,48,49,50] (Figure 2). Although TRPA1’s presence and role is most widely known in the peripheral nervous system, TRPA1 receptors have also been discovered in the central nervous system with distribution in the cortex, caudate nucleus, putamen, globus pallidus, substantia nigra, hippocampus, cerebellum, amygdala, and hypothalamus [51] (Figure 2). The extent of TRPV1’s function in the CNS has yet to be fully determined; however, its wide tissue distribution certainly suggests a pleiotropic role.

### 3.2. TRPV1 and TRPA1: Structure and Function in Pain

Nociception at its origin involves the activation of unmyelinated C-fibers and myelinated Aσ-fibers by noxious stimuli [52]. The nociceptive pathway continues to encompass the process of acute pain perception and response, involving the transduction of noxious stimuli by way of somatosensory processes and release of chemical mediators by neurons in the periphery, consequent transmission, followed by modulation and sensitization [52,53]. Transmission describes the process in which this pain signal is carried from sites of tissue stimulation to brain regions responsible for pain perception [54]. In normal pain transmission, an influx of sodium and calcium ions leads to local depolarization; when a threshold is reached, an action potential is propagated via non-synaptic ion channels along the axon to the dorsal root ganglion, followed by the dorsal horn [52]. 

The TRPV1 receptor is perhaps the most extensively elucidated member of the TRP superfamily, first cloned by expression-cloning screening in 1997 [43]. The TRPV1 channel is predicted to be a homotetrameric complex whose structure is akin to many other ion channels, with each subunit containing six transmembrane domains and a short hydrophobic sequence between the fifth and sixth domains, forming a functional pore [43,55,56,57]. Analysis of TRPV1 structure has suggested the importance of ankyrin repeat domains (ARDs) contained in the cytosolic NH_2_ and COOH termini in desensitization of the channel; these ARDs serve as binding sites for calmodulin and ATP, with a modulatory effect being more emphatically apparent in the calmodulin-binding site in the NH_2_ terminus [22,58]. TRPV1, also known as the capsaicin/heat receptor, has long been known to be responsive to noxious heat stimuli, low pH, and vanilloid agonists (e.g., capsaicin), as well as a variety of other lipids, leading to the sensation of pain and temperature regulation [43,57,58,59,60,61,62]. Recent studies have shown that competitive antagonists of various subfamilies of TRP channels attenuate thermal hyperalgesia from inflammatory conditions [31]. Mice lacking certain functional TRP genes, like TRPV1, exhibited a dramatic attenuation of thermal hyperalgesia in response to inflammatory stimuli [31,32,33,34]. Desensitization of TRPV1 has been known to yield an analgesic effect due to its role in pain conductance, which has made TRPV1 a prime target in the investigation of novel therapies. While TRPV1 stimulation by capsaicin and its analogs is well documented [63,64,65], its interaction with caffeine remains an area of active research.

Alongside TRPV1, TRPA1 receptors have been distinguished as important modulators in pain. The TRPA1 channel was first cloned in 1999 and, similar to other TRP channels, consists of a pore-forming homotetrameric structure, but is distinguished by 14–19 ankyrin repeats at the NH_2_-terminus [51,66,67]. TRPA1 has been popularly referred to as ANKTM1 in the literature due to its numerous ankyrin repeats, which provide the channel its ability to bind cytoskeletal proteins via protein–protein interactions, channel insertion and plasma membrane regulation, and unique thermal and chemical sensitivities [66,67,68]. This portion comprises approximately 64% of the channel, and additionally contains cysteine and lysine residues with roles in disulfide bridging and channel activation, as well as an EF-hand domain with the primary action of calcium binding [51,69]. Of note, Moparthi et al. demonstrated increased heat and cold sensitivity in human TRPA1 without N-terminus ankyrin repeat domains, suggesting a greater role of C-terminal domains in TRPA1 sensitivity than previously thought [70]. Activation of TRPA1 leads to Ca^2+^ influx with outward rectification due to covalent modification of cysteine residues [71], leading to a variety of downstream effects. 

Human TRPA1 structure has been demonstrated via cryoelectron microscopy to possess positively charged domains that interact with various irritants, including isothiocyanates, thiosulfinates, and α,β-unsaturated aldehydes [51,72]. In the lab, Macpherson et al. demonstrated the activation of TRPA1 by noxious compounds via covalent modification of reactive cysteine residues, validating the activation of the channel by both electrophilic and non-electrophilic compounds [71]. TRPA1 is closely related to TRPV1 in terms of distribution in skin-innervating nociceptive neurons and activation by noxious stimuli [73] and is responsive to an incredibly wide spectrum of activating stimuli from exogenous and endogenous irritants, low pH, osmotic pressure, and extremes in temperature [47,73,74,75,76,77,78]. Of note, it is estimated that TRPA1 is present in 30–50% of TRPV1-positive neurons, and is rarely found in TRPV1-negative neurons, highlighting their close association and shared role in nociception [79].

TRPA1 has been demonstrated to play key roles in acute and inflammatory nociception, neuropathic pain, cancer-related pain, migraine headache, and dysfunctional pain by various mechanisms [24,31,32,47,51]. Heber et al. demonstrated via intraepidermal injections of TRPA1 agonist and antagonist that isolated stimulation of TRPA1 produces pain sensation independent of other factors, affirming its role in acute pain generation [47]. Beyond painful sensory functions in the skin, TRPA1 possesses roles in inflammatory, neuropathic, and visceral pain. The responsiveness of TRPA1 to endogenous mediators of inflammation and tissue damage, including prostaglandins, hydrogen peroxide, and other byproducts of oxidative stress, makes it a key player in exploring novel treatment options for inflammatory pain [22,50]. In particular, beyond its chemical and physical activators, TRPA1 has been shown to be modulated by proalgesic and proinflammatory agents, such as bradykinin activating phospholipase C, contributing to excitation and sensitization of nociceptive neurons [72,80].

## 4. Caffeine in Analgesia

### 4.1. Mechanism of Action

Caffeine has primarily been studied for its pain-relieving effects through the competitive inhibition of adenosine receptors (ARs), particularly A1, A2a, and A2b subtypes [81,82,83]. These G-protein-coupled receptors are classified based on differential coupling to adenylyl cyclase, either Gi/o proteins or Gs/o proteins [84]. Activation of ARs leads to the regulation of cyclic AMP levels and has been shown to be capable of a variety of downstream effects in human and animal models, including changes in activity of protein kinase A (PKA), protein kinase C (PKC), phospholipase C (PLC), cardiac K^+^ channels, voltage-gated Ca^2+^ channels, and the mitogen-activated protein kinase (MAPK) pathway [83,84,85]. Adenosine receptors modulate the physiological activity of the neurotransmitter, adenosine, which has been shown to play roles in brain functions such as endogenous sleep regulation and drowsiness, neurocognition, memory, and energy metabolism; adenosine is also involved in the development of neurologic and neurodegenerative diseases [85,86,87,88,89]. Recent studies have also pinpointed adenosine receptor activation as a potential target in the management of acute and chronic pain [90,91,92,93,94]. Caffeine can reduce pain through several mechanisms: improving drug absorption through lower gastric pH and increased gastric blood flow, downregulating cyclooxygenase-2(COX-2), antagonizing the adenosine receptor, and impacting emotional states that can contribute to the perception of pain [95].

### 4.2. Caffeine: Adjuvant Analgesia

The physiological effects of caffeine have been described in the literature, including its adjuvant effect in the role of analgesia [94,96]. The term adjuvant in this literature is used to describe a substance which, when used in conjunction with a pharmacologic agent, enhances or modifies the agent’s effects [97]. Compared to the use of ibuprofen alone, combining it with caffeine enhances pain relief [98]. This combination, encompassing the standard dose and 100 mg caffeine, is roughly equivalent to consuming one 200 mg ibuprofen tablet and a cup of moderately strong coffee, coffee tablets, tea, cola drinks, or energy drinks [99]. For decades, caffeine has been added to multiple routinely used analgesics, including aspirin, phenacetin, acetaminophen, paracetamol, and salicylamide [81,82,100]. 

The widespread usage of caffeine in analgesic medications is perhaps a testament to its utility and has prompted studies into its efficacy as an adjuvant, with a small significant benefit in analgesic effect with the addition of caffeine seen in meta-analyses [95,100]. At dosages of 200 mg to 300 mg, it has been shown to demonstrate anti-inflammatory and antioxidant action through reducing oxidative stress, decreased levels of proinflammatory cytokines such as tumor necrosis factor (TNF) and interleukin (IL), and reduced cell membrane lipid peroxidation [101]. Lipton et al. demonstrated a significant analgesic effect in the treatment of acute primary headache and migraine headache with doses of caffeine greater than 100 mg adjuvant to analgesic medications of ibuprofen, acetaminophen, and acetylsalicylic acid [102]. It should be noted that the use of caffeine as adjuvant therapy should be limited to acute pain management due to the risk of physical tolerance, overuse headache, and withdrawal symptoms if discontinued abruptly [103].

### 4.3. Caffeine: Surgical Uses

Recent studies emphasize the role of caffeine in postoperative care. In terms of anti-inflammatory effects, the use of caffeine-containing analgesics (300 mg acetaminophen and 20 mg caffeine) is significantly more efficient than using those containing codeine (300 mg acetaminophen and 20 mg codeine capsule). Conversely, for analgesia, codeine-containing analgesics are significantly more effective than caffeine-containing ones [104]. Thus, the use of caffeine is considered useful as an adjuvant postoperatively for its anti-inflammatory effects but may be limited in its ability to independently provide analgesia.

Caffeine is also useful during anesthesia emergence due to its known effects on wakefulness and arousal. Intravenous caffeine has been shown to accelerate emergence from isoflurane, even at high levels, in healthy adult males with no signs of adverse effects [105]. Given the lack of specific anesthesia reversal therapies, caffeine offers a potentially low-cost and safe approach to improve post-anesthesia care. However, further studies with a larger sample size are needed to assess its risks and benefits [105].

## 5. TRP and Caffeine

**Figure 3:**
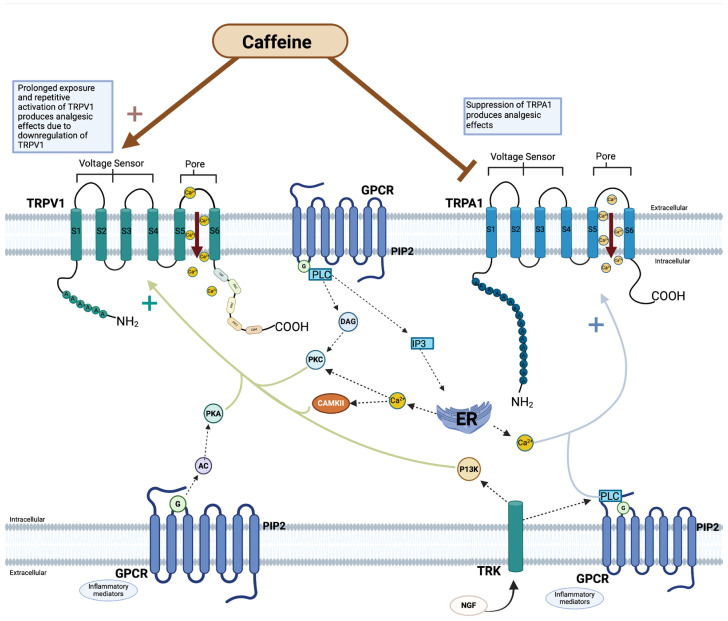
Caffeine modulation of TRPV1 and TRPA1. Visualization of the effect of caffeine on transient receptor potential (TRP) channels—TRPV1 and TRPA1 subfamilies of transient receptor potential (TRP) channels. Both subfamilies have a pore between the fifth and sixth transmembrane that yields nonselective calcium ion permeability. Caffeine is theorized as an agonist of TRPV1, but with prolonged exposure and activation, causes receptor downregulation. Caffeine is postulated to act as an antagonist against TRPA1. Downstream processes are regulated by a variety inflammatory mediators (e.g., bradykinin, adenosine triphosphate, prostaglandin, serotonin) and secondary messengers, to either activate or desensitize TRP channels to stimuli [102]. The activation of the intracellular pathways leads to calcium release from the endoplasmic reticulum, causing an increase in Ca^2+^/calmodulin-dependent protein kinase II (CAMKII) and PKC. G-protein-coupled receptors (GPCR) are transmembrane proteins with 7 transmembranes that tune cellular functions by the activation of G-protein-dependent cascade and transducing extracellular signals. The G-protein cascade and transduction of extracellular signals mediates TRP channel sensitization and activation [103]. (Created with BioRender.com).

Mammalian subfamilies of TRP have been shown to play important roles in both non-excitable and excitable cell types, demonstrating responsiveness to a wide variety of cellular signals [95]. One such signal includes trimethylxanthine, a psychotropic alkaloid colloquially known as caffeine [96]. However, caffeine is known to affect various receptors in the human body, including TRP channel subtypes TRPV1 and TRPA1 (Figure 3). Caffeine has been demonstrated to increase intracellular Ca^2+^ concentrations by mobilization of calcium stores in intracellular organelles and enhanced influx of extracellular Ca^2+^; the literature describes caffeine’s effect on Ca^2+^ influx via voltage-gated calcium channels (VGCCs) most extensively, compared to other channels [97,98,99]. However, discovery of TRP channels with calcium permeability prompts a more comprehensive understanding of the interaction between caffeine and TRP channels. Notably, Masuho et al. demonstrated a disappearance in caffeine-induced Ca^2+^ influx in the presence of a phospholipase C (PLC) inhibitor in STC-1 intestinal cells, while Nagatomo et al. demonstrated a similar disappearance in caffeine-induced Ca^2+^ influx in the absence of extracellular calcium stores in STC-1 intestinal cells [100,101]. These observations suggest involvement of a caffeine-activated channel sensitive to PLC inhibition in calcium conductance, rather than solely a Gq-sensitive channel as was previously thought. One possibility of this caffeine-activated channel is theorized to be TRP. 

### 5.1. TRPV1 and Caffeine 

When activated by vanilloids including capsaicin, TRPV1 functions as a nonselective cation channel enabling influx of calcium and sodium [104]. An influx of ions through TRPV1 channels transduces noxious stimuli into locally spreading membrane depolarizations, carrying action potentials to the central nervous system [105]. In the case of capsaicin, TRPV1 channels are initially activated, leading to temperature and pain sensations; however, with prolonged and repetitive activation, TRPV1 channels undergo desensitization via calcium cytotoxicity and nociceptor ablation [105,106]. By this mechanism, excess activation of TRPV1 channels produces an analgesic effect (Figure 3). 

Injection or topical application of capsaicin at high doses, either in a single high dose or repetitive administration, abolishes thermal pain in skin receptors and produces an analgesic effect in cancer-related and arthritic pain [107,108,109]. In general, prolonged exposure of TRPV1 to agonists, including the vanilloid resiniferatoxin, which holds 1000-fold potency over capsaicin, induces endocytosis and lysosomal degradation in TRPV1 receptors [108,110]. Agonist-induced receptor internalization demonstrates clathrin- and dynamin-independent endocytosis triggered by Ca^2+^ influx through TRPV1 [108].

Caffeine is suggested to interact with TRPV1 by acting as an activator and causing a caffeine-induced Ca^2+^ influx across the plasma cell via the TRPV1 channels [108,111]. In a study conducted by Daher et al., caffeine-induced Ca^2+^ transients were measured via single-cell microfluorimetry in rabbit nodose ganglion neurons (NGNs), with significant attenuation seen with application of TRPV1 antagonism; this suggests a caffeine-induced Ca^2+^ influx across the plasma membrane via TRPV1 channels, which affirms caffeine as an activator of TRPV1, and suggests the possibility of caffeine-induced receptor downregulation [111]. 

This is reaffirmed by Cleland et al., in which the presence of 10 µm caffeine provided protection against ethanol-induced constriction of rat middle cerebral artery (MCA), but not in the presence of a selective TRPV1 blocker. The most likely explanation presented by the study for the caffeine–TRPV1 interactions observed was that caffeine induces activation of the TRPV1 receptor [112]. Assuming similar activation of TRPV1 with caffeine as capsaicin, downregulation of TRPV1 channels by modulating expression of the channel might present a potential mechanism for caffeine’s adjuvant analgesic effects.

### 5.2. TRPA1 and Caffeine

Human TRPA1 channels are suggested to be acted upon by caffeine in an antagonistic manner as caffeine directly interacts with and influences the channels’ activity [101,113]. Nagatomo et al. demonstrated that while caffeine induces an activation response in mouse TRPA1 channels, the same dose and method of application of caffeine induces suppression in human TRPA1 channels [101]. In the same study, it was affirmed that caffeine itself directly influences TRPA1 activity, rather than indirect activation by way of Ca^2+^ influx [101].

Bianchi et al. evaluated preservation of TRPA1 morphology and activation across species by fluorescent Ca^2+^ influx assay using transiently transfected human embryonic kidney 293-F cells. This study found that while human and rhesus monkey TRPA1 channels exhibited similar responses to caffeine, rodent TRPA1 responded in a pharmacologically distinct fashion unlike either humans or rhesus monkeys [114]. Indeed, species-specific difference was observed in TRPA1 sensitivity to the electrophilic, thioaminal-containing compound CMP1 as inducing activation in mice and blockade in humans [115]. Nagatomo et al., in a separate study, attributed this to genetic differences, specifically in amino acids 231–287 of the cytoplasmic N-terminus of mouse TRPA1 (mTRPA1); point mutation of methionine 268 proline (Met268Pro) induced a change in caffeine activation of mTRPA1 to suppression [116]. This provides a potential molecular target for the attribution of species-specific differences in caffeine-induced TRPA1 activation (Figure 3).

Further studies are indicated to fully explicate the interplay between caffeine and TRPA1 as an emerging target for novel pain control drugs. While these findings confirm the significance of caffeine as a regulator of TRPA1, the findings described above perhaps indicate a need for a shift in the current subjects being studied and methods of application to determine the true pharmacological impact of caffeine in human TRPA1, which may be translated into future therapeutic interventions in pain.

Transient receptor potential vanilloid 1 (TRPV1) is known to play a role in acute nociceptive pain sensation and temperature regulation. Caffeine is an agonist of TRPV1; prolonged exposure and repetitive activation of TRPV1 produces analgesic effects due to the downregulation of TRPV1. Transient receptor potential ankyrin 1 (TRPA1) is known to play a role in acute and inflammatory nociception, neuropathic pain, cancer-related pain, migraine headache, and dysfunctional pain. Caffeine is an antagonist, causing suppression of TRPA1 channels, which produces analgesic effects. Adenosine receptors (A1, A2a, and A2b) play a role in sleep, cognition, memory, and hyperalgesia. Caffeine is a competitive inhibitor of adenosine receptors, producing an adjuvant analgesic effect, wakefulness, prolonged sleep latency, and arousal. 

## 6. Discussion

**Figure 4:**
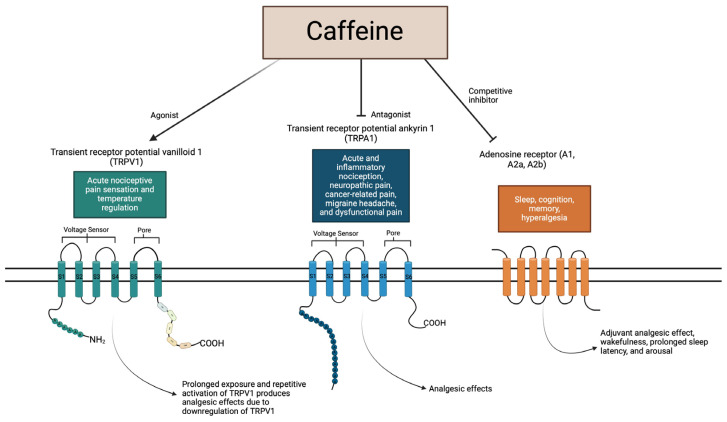
Summary of caffeine interplay with receptors.

Recent interest in TRP has revealed a myriad of molecular modulators, among them the commonly consumed trimethylxanthine, caffeine. Caffeine largely exerts its psychoactive properties by antagonism of adenosine receptors, inhibiting the somnolence and relaxation of the neurotransmitter adenosine and causing increased alertness and arousal (Figure 4). 

Of note, it should be mentioned that there is uncertainty in whether caffeine interaction with adenosine receptors directly leads to any influence on TRP activity. While the interaction between caffeine and adenosine receptors is well established, very little is known about potential interactions of TRP channels with adenosine receptors; current evidence demonstrates the importance of calcium ion currents to adenosine receptor functionality, but further evidence is needed to link TRP modulation to adenosine receptor function [117,118,119]. Further studies exploring whether TRP channels modulate the activity of adenosine receptors could provide valuable insights into the complex mechanisms underlying caffeine’s actions and open avenues for novel therapeutic interventions targeting these receptors.

Dose-related adverse effects of caffeine must also be recognized as a limiting factor to its therapeutic use. Caffeine intake has been shown to produce harmful effects in the human body when exceeding 450 mg per day, largely due to antagonism of inhibitory adenosine receptors causing a state of excitatory dysfunction [120]. Acute adverse effects of caffeine may include nausea, irritability, restlessness, and palpitations, posing danger in certain predisposed individuals [121]. This is dependent on individual variation with recorded instances of fatality from reported caffeine consumption below 400 mg [122]. The elicited response to caffeine is also seen regardless of the method of application; common sources of caffeine include caffeine tablets, coffee, cola drinks, energy drinks, and plain chocolate [123]. Further studies in the lab will be required to determine the dosage and mode of delivery (e.g., topical, injection) of caffeine that may be safe and effective in TRPV1 desensitization, as well as the timing and degree of evoked ionic changes.

Regarding caffeine and TRPV1, caffeine is theorized to have an agonistic effect on TRPV1 channels, leading to eventual receptor desensitization and downregulation. However, current TRPV1 agonists have exhibited varying capability to produce shifts in ionic selectivity, which may lead to a variable analgesic benefit. This can be seen in the difference in elicited response between caffeine and capsaicin. In some randomized control trials studying resistance training, oral capsaicin has been shown to produce a reduction in rate of perceived exertion (RPE), theorized to be due to an increased discomfort threshold due to the analgesic effect [124,125]. However, caffeine by the same oral route has failed to produce a similar significant reduction in RPE in other studies, either when used alone or in conjunction with capsaicin [126,127]. Activation of TRPV1 leading to desensitization is dependent on potency of agonist, agonist concentration, time, and administration site [108,128]. As aforementioned, characteristics of cytosolic NH_2_ and COOH termini also contribute to the likelihood of desensitization of TRPV1 [129,130]. For example, increased evoked changes in ionic selectivity have been seen with phosphorylation of S800 in the NH2 terminus by way of protein kinase C [131]. There is also variability in length of nociceptor refractory periods, which can last from minutes to months [108].

Regarding existing TRPV1 agonists, therapies using pungent compounds such as capsaicin are dose-limited due to pain upon initial application prior to relief; ligands such as olvanil and MRD-652 have been developed and have shown promising efficacy in animal models while minimizing initial pain response [132]. Resiniferatoxin, previously mentioned in this review for its potent agonist activity capable of desensitizing TRPV1 response, has been named as a “molecular scalpel” for chronic pain relief, especially in bone and arthritic pain deriving from bony metastases, osteosarcoma, and osteoarthritis [110,133,134]. However, attempts of therapies utilizing TRPV1 antagonist compounds have had less measurable success in alleviating inflammatory and neuropathic pain in animal models, with varied results [135]. While antagonist compounds such as JNJ-39439335 (mavatrep) and SB-366791 have shown promise in alleviating osteoarthritic pain and dental pain, respectively, compounds such as NEO6860 were unable to produce significant results when compared to placebo [132,134,136,137,138]. Given the mixed results of TRPV1 antagonists and the promising efficacy of TRPV1 agonists, it may be that compounds with agonist activity such as caffeine may produce more reliable results in treating pain through modulation of TRPV1.

Regarding caffeine and TRPA1, caffeine is theorized to act as an antagonist of TRPA1 channels, directly suppressing the pain response activation of TRPV1 elicits. Caffeine as an antagonist of human TRPA1 channels presents a promising avenue for novel treatments in pain, as suppression of channel activity yields an analgesic effect; further, antagonism of TRPA1 by various ligands studied thus far have not presented any significant concerns to safety, suggesting this to be a favorable pathway for novel pain drugs [113]. However, there is a relative scarcity of data surrounding the ligand–host interaction between caffeine and TRPA1, and further studies in the laboratory are needed to definitively evaluate its utility in the treatment of pain disorders. 

The development of TRPA1 drug therapies is an area of active research. TRPA1 antagonism in molecular models has shown to be capable of alleviating hyperalgesia, neuropathic pain, arthritic pain, postoperative pain, migraine headache, and visceral pain [113,139]. The antagonist compounds HC-030031, a xanthine derivative, and TCS-5861528 are capable of preventing or delaying migraine headache through attenuation of inflammation and hypersensitivity [140,141]. Chembridge-5861528, another xanthine antagonist of TRPA1, was found to attenuate mechanical hypersensitivity and exhibited potency 10 times that of HC-030031. The efficacy of xanthine derivatives may represent a promising future for the incorporation of the methylxanthine, caffeine, into TRPA1 therapies.

## 7. Conclusions

In this paper, we outlined a brief review of the current evidence as to the role of caffeine in analgesia, the role of TRP in pain and analgesia, as well as what is known regarding the interaction of caffeine with TRPA1 and TRPV1. In summary, caffeine has been demonstrated to interact with TRP channels both directly and indirectly, most notably the TRPV1 and TRPA1 channels. Caffeine regulation of these channels may represent potential for future therapies, as these receptors play significant roles in pain disorders. The modulation of TRP by caffeine may be either suppression or activation, and is dependent on TRP channel subtype, dosage of caffeine, method of administration, genetic differences, and recipient species. 

However, the exact molecular mechanism of caffeine binding to TRP channels has yet to be demonstrated in the lab. Although caffeine is known to influence the activity of TRP channels, there is a relative scarcity of evidence regarding the binding site, mediators, and downstream effects of caffeine with TRP. Caffeine has adjuvant analgesic properties, and the recently established role of TRP in pain and analgesia prompts the investigation of whether caffeine interaction with TRP is responsible for some of its analgesic effect. Further investigation into the ligand–receptor relationship between caffeine and TRPs will require an expansion in study methods, subjects, and mode of administration. Findings thus far are largely limited to the animal model; more data are needed to fully validate the clinical utility of caffeine to provide therapeutic benefits for patients with pain disorders through the modulation of TRPs.

## Data Availability

No new data were created in the writing of this review. All utilized data were obtained via the PubChem and/or Google Scholar public databases.
